# Interoperability of text corpus annotations with the semantic web

**DOI:** 10.1186/1753-6561-9-S5-A2

**Published:** 2015-08-06

**Authors:** Karin Verspoor, Jin-Dong Kim, Michel Dumontier

**Affiliations:** 1 Dept of Computing & Information Systems, The University of Melbourne, Melbourne, Australia; 2 Database Center for Life Science, Research Organization of Information and Systems, Kashiwa, Japan; 3 Stanford Center for Biomedical Informatics Research, Stanford University, Stanford, CA, USA

## Summary

This paper explores the adaptation of the PubAnnotation model with recent more general proposals for the representation of annotations in the Semantic Web, referred to here as the Open Annotation model and the focus of the W3C Web Annotation Working Group. We argue that interoperability with standards under development for text annotation on the web, and with recent proposals related to nanopublications, will have benefits for the use and consistency of linguistically annotated text corpora.

## Introduction

Formal annotation of language data is an activity that dates back at least to the classic work of Kucera and Francis on the Brown Corpus [1]. It further is a general scholarly activity by which scholars organize existing knowledge and facilitate the creation and sharing of new knowledge. Annotation is also becoming increasingly pervasive in the context of social media. Recognition of the widespread importance of annotation has resulted in recent efforts to develop standard data models for annotation [2-4], specifically targeting Web formalisms in order to take advantage of increasing efforts to expose information on the Web, such as through Linked Data initiatives (http://linkeddata.org). The WWW Consortium (W3C) has formed the Web Annotation Working Group (http://www.w3.org/annotation/) to develop specifications for a Web annotation architecture.

In this paper, we propose the adoption of general semantic web-oriented annotation proposals for text annotation in the context of text corpora intended for use in developing Biomedical Natural Language Processing (BioNLP) solutions. We specifically look at adapting the current PubAnnotation format [5] for compatibility in relation to those proposals. We propose a representation of an annotated corpus in terms of the data models under development in the broader scholarly annotation community, and develop a translator from the existing PubAnnotation JSON format to the Open Annotation model.

This generalization of the model is particularly pertinent to collaborative annotation scenarios; exposing linguistic annotations in the de facto language of the Semantic Web, the W3C's Resource Description Framework (RDF), provides several advantages that we have previously described [6]. We further demonstrate that the model can be integrated with the nanopublications model [7,8], facilitating their use in a growing set of data publication tools [9].

## Annotation models

### PubAnnotation

PubAnnotation is an annotation repository, that also provides a web services interface exposing the underlying texts and associated annotations [5]. This interface makes use of a simple JSON format that directly associates a span of text to a particular concept string.

### Open Annotation model

The W3C Web Annotation working group will base its proposals in the prior Annotation Ontology [2] and Open Annotation Collaboration [3] models. Each of these models in turn incorporates elements from the earlier Annotea model [10]. We refer to this model as the Open Annotation model (OpenAnn) [4], and adopt it for our target representation.

#### High-level model for scholarly annotation

The basic high-level data model of the two primary Open Annotation models defines an *Annotation *as an association created between two elements, a *Body *or content resource and (one or more) *Target *resources. The annotation provides some information about the target through the connection to the body. For instance, an annotation may relate the token "apple" in a text (the target of the annotation) to the concept of an apple, perhaps represented as WordNet [11] synset "apple#1" (the body of the annotation).

**Figure 1:**
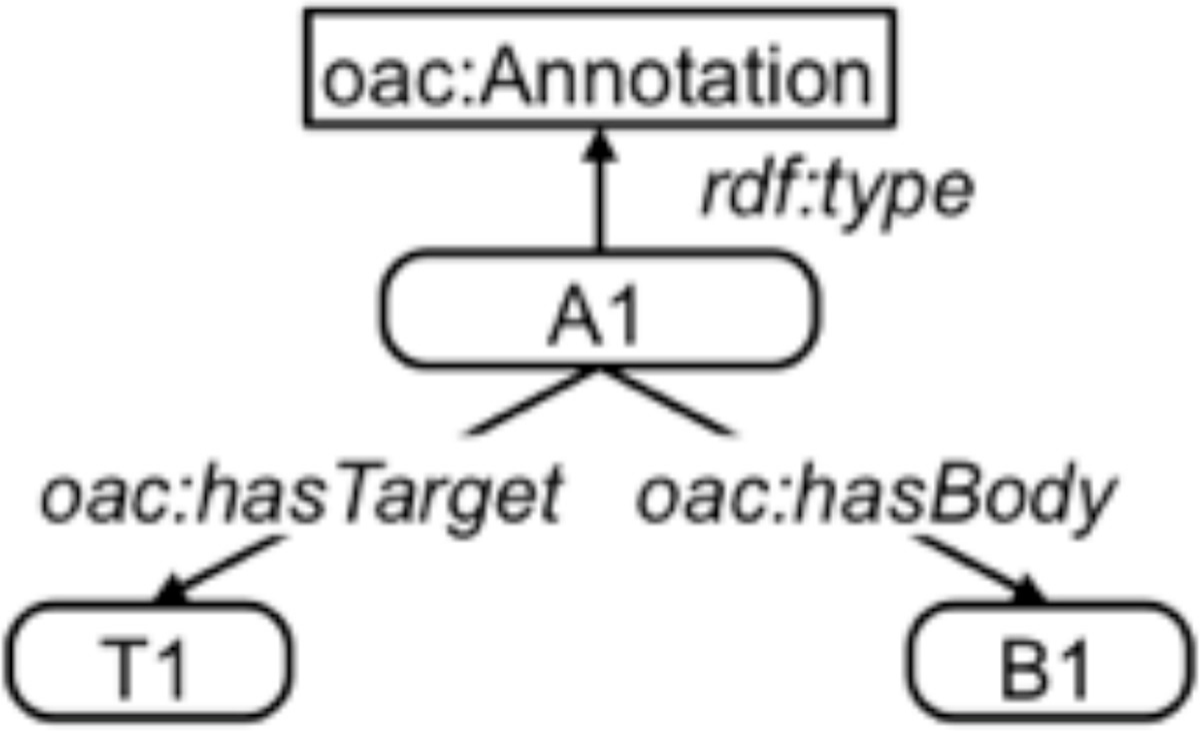
**Base model for OpenANN**.

Figure 1 shows the base model defined in the OpenAnn model. The model, following linked data principles, assumes that each element of an annotation is a web-addressable entity that can be referenced with a URI.

Annotations can be augmented with meta-data, e.g. the author or creation time of the annotation. The model allows for each element of the annotation - the annotation itself, the target, and the body - to have different associated meta-data, such as different authors.

#### Graph Annotations

The initial use cases for Open Annotation focused on single target-concept relationships, formalized as an expectation that the body of an Annotation be a single web resource, represented as a URI. However, to accommodate more complex bodies, a set of RDF statements can be captured in a construct known as a *named graph *[12]. The named graph as a whole has a URI. We propose to bundle all *Body *content into a named graph, so that both simple (e.g., entity) annotations and more complex (e.g., event) annotations can be captured in a consistent representation.

This extension enables complex semantics to be associated with a resource, as well as supporting fine-grained tracking of the provenance of compositional annotations. These developments make possible the integration of linguistic annotation with the scholarly annotation models [13].

## Representing PubAnn in OpenAnn

As an example of the use of OpenAnn for PubAnn, we transform a PubAnn JSON statement for an entity into OpenAnn. The PubAnn statement

{"id":"T13","span":{"begin":1304,"end":1309},"obj":"Protein"}

is represented in OpenAnn as the following set of RDF statements:

# The basic annotation structure

:provenance1 {

<PubMed-­‐1134658-­‐Ann1> a oa:Annotation.

<PubMed-­‐1134658-­‐Ann1> oa:serializedBy <http://pubannotation.org>.

<PubMed-­‐1134658-­‐Ann1> oa:hasTarget <PubMed-­‐ 1134658-­‐0-­‐SR1>.

<PubMed-­‐1134658-­‐Ann1> oa:hasBody <PubMed-­‐ 1134658-­‐0-­‐T13>.

<PubMed-­‐1134658-­‐Ann1> oa:motivatedBy oa:tagging.

<PubMed-­‐1134658-­‐Ann1> prov:generatedOn "20141111".

<PubMed-­‐1134658-­‐0-­‐T13> prov:derivedFrom<PubMed-­‐1134658-­‐0-­‐SR1> . }

# The body (content) of the annotation.

# A named graph.

<PubMed-­‐1134658-­‐0-­‐T13> {

<PubMed-­‐1134658-­‐0-­‐SR1> sio:refers-­‐to genia:Protein . }

# The target of the annotation.

<PubMed-­‐1134658-­‐0-­‐SR1> a oa:SpecificResource;

oa:hasSource <http://pubannotation.org/docs/sourcedb/PMC/sourceid/1134658/divs/1.txt> ;

oa:hasSelector <PubMed-­‐1134658-­‐0-­‐S1304-­‐1309>.

# A selector for a location within the text resource.

<PubMed-­‐1134658-­‐0-­‐S1304-­‐13099> a oa:TextPositionSelector ;

      oa:start 1304 ;

      oa:end 1309.

We also extend our representation to be compatible with nanopublications (http://www.nanopub.org/guidelines) [7,8], a community standard for encapsulating assertions with their provenance into a portable digital object, by defining the annotation body to be the assertion of a nanopublication.

:np {

np:has-­‐assertion <PubMed-­‐1134658-­‐0-­‐ T13>;

a np:Nanopublication . }

The above approach can be similarly applied for capturing relational or event semantics as the body of an annotation, by encapsulating a set of triples representing the event within a named graph. We leave such examples for a more in-depth paper.

## Discussion

The adoption of the Open Annotation formalism for representing annotations over textual corpora brings those annotations into the realm of the semantic web, enabling consistent specification of annotation content, provenance, and meta-data in terms of resolvable and reusable ontology concepts. It will allow annotations generated by different systems or individuals over the same documents to be more easily integrated, compared and contrasted. It further ensures interoperability of corpus annotations with components for authoring, sharing, and displaying annotations in browsers and other technical systems that will be developed through the broader efforts of the W3C, including digital publishing tools (*cf*. the Domeo annotation toolkit for the precursor of the Open Annotation model [14]).

Nanopublications seem to be a particularly apt choice for structuring OpenAnn text annotations in the biomedical domain. Using nanopublications, the assertion, provenance, and metadata for a PubAnnotation are clearly demarcated into named graphs, which can retrieved, validated, and viewed by a growing set of data publication tools [9].

Furthermore, nanopublications are being used in an increasing number of biomedical resources to represent factual assertions and their provenance, and a number of tools are being developed specifically to work with nanopublications (e.g., the NanoBrowser http://nanobrowser.inn.ac/). They have been used for incentivizing the publication of human variation data [15], capturing claims [16] and scientific discourse [17], and publishing text-mined associations [18]. Bringing together Open Annotation with nanopublications offers substantial opportunities for access to and reuse of text annotations in combination with information derived from structured databases.

## Conclusions

We have introduced a proposal for the representation of text annotations in terms of the Open Annotation model, and demonstrated how it could be applied to the current PubAnnotation JSON format. We structured our model to also be compatible with nanopublications, in order to enable integration of text annotations with information derived from curated databases. The result is a representation for text annotation on the web that is interoperable with the framework of two increasingly relevant semantic web models.
